# Breast metastasis of a lung carcinoma

**DOI:** 10.1259/bjrcr.20210142

**Published:** 2021-12-14

**Authors:** Ralph Khoury, Margot Bucau, Alexandra Bizot, Antoine Khalil

**Affiliations:** 1 Radiology, Hopital Bichat-Claude-Bernard, Paris, France; 2 Anatomo-Pathology Department, Hopital Bichat-Claude-Bernard, Paris, France; 3 Thoracic-Oncology Department, Hopital Bichat-Claude-Bernard, Paris, France; 4 Paris University, Paris, France

## Abstract

Breast metastasis is a rare phenomenon (0.2–1.3%)^1^ compared to primary breast lesions. Several neoplasms have been reported to metastasize to the breast such as melanoma, lymphoma and lung cancer. In this article, we report a case of breast metastasis of lung cancer confirmed by biopsy and immunohistochemistry with CT and ultrasound imaging.

## Clinical presentation

A 35-year-old female presented to her family doctor for a self-palpable cervical node and cough of 2-month duration along with dyspnoea upon exertion for the past month. On clinical examination, she was hypertensive (210/80 mmHg), tachycardic (116 bpm beats per minute), and were palpated a left cervical node and a left breast nodule. She reported no headache, no nausea and no loss of weigh. She had no previous medical history and had been smoking for 15 years (15 pack-year). She was referred to our hospital for imaging evaluation.

**Figure 1.**
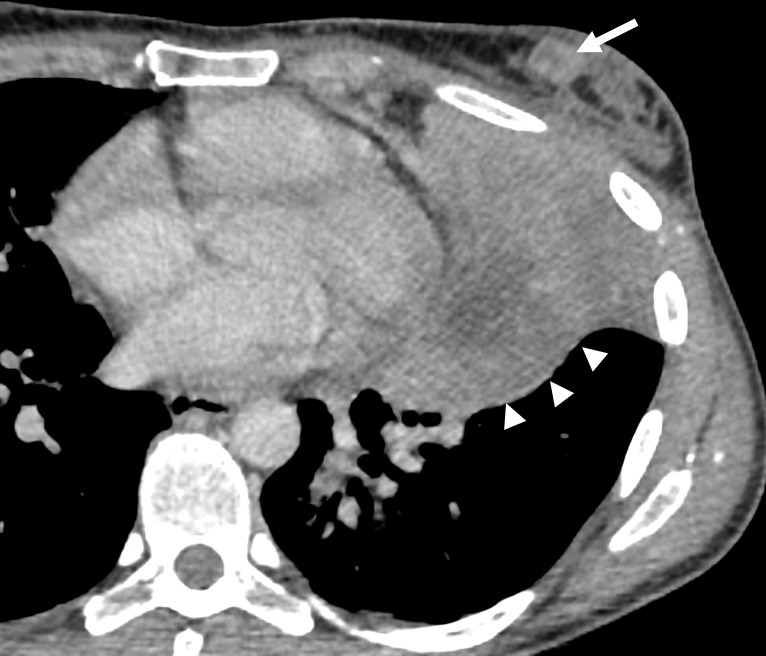
Axial CT-scan showing the breast nodule (arrow) and the lung lesion (arrowheads).

A thoracic, abdominal and pelvic CT scan showed a large lung consolidation involving the entire left upper lobe (Figure 1, arrowheads) highly suggestive of lung cancer. Bronchial biopsy confirmed a non-small cell lung carcinoma, with modification of Ros 1. (Reactive oxygen species 1).

A brain MRI was also done showing five millimetric metastatic brain lesions.

**Figure 2.**
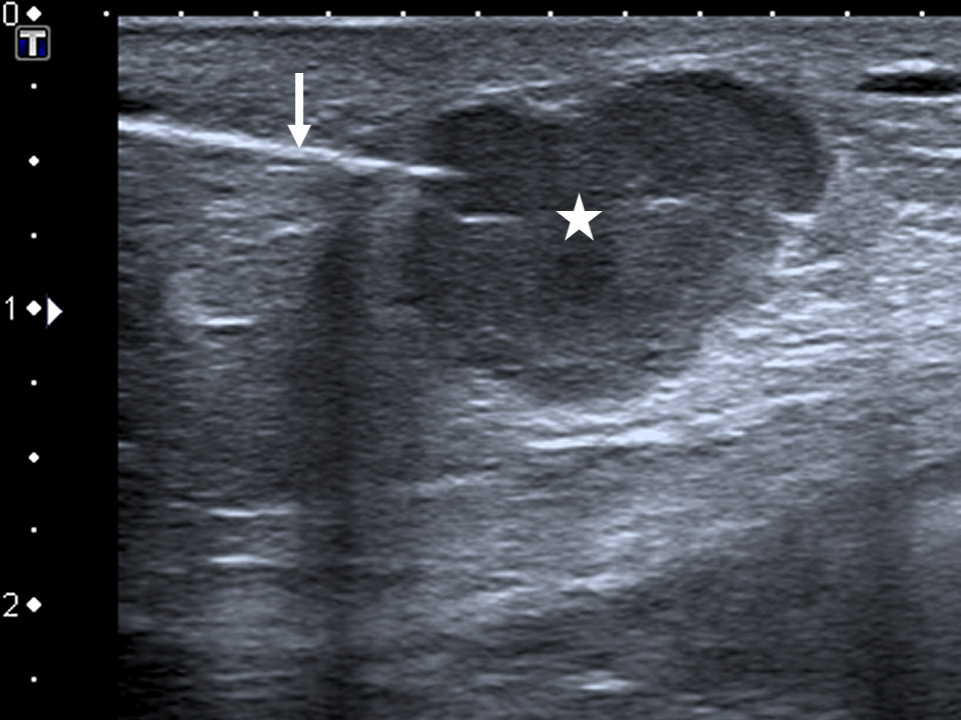
Ultrasound image of the left breast showing the microlobulated hypoechoic nodule (star) with posterior enhancement and the biopsy needle (arrow).

However, an additional finding could be seen in the left breast, a small enhancing nodule of the inner lower quadrant (Figure 1, arrow). Subsequently, we decided to do an ultrasound which showed an irregular, circumscribed, microlobulated, hypoechoic nodule, with no posterior features, measured at 15 mm (ACR BIRADS 4). The context of cancer and the microlobulated aspect of the nodule justified breast biopsy that was done later on with a 14G co-axial system (Bard^®^,Mission^®^) (Figure 2).

**Figure 3.**
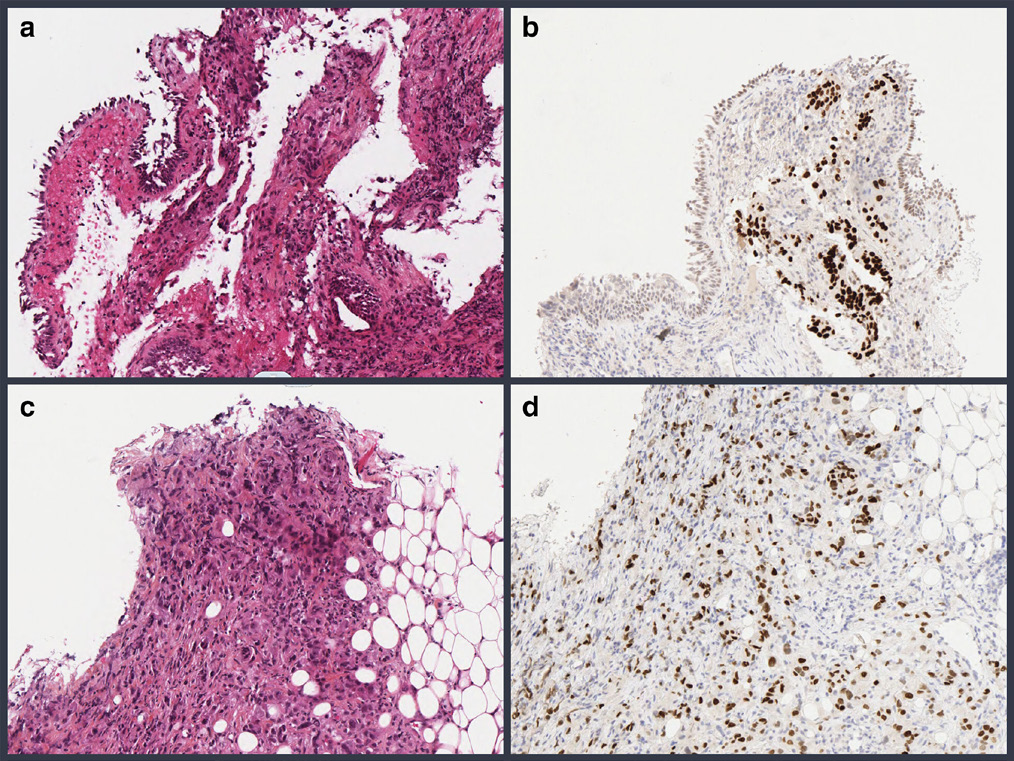
Figure grouping the HES stains and the anti-TTF1 immunostaining of the lung and breast specimen. (a) HES staining, magnification x100: proliferation of cells in the chorion with atypical large nuclei under a normal bronchial epithelium. The cells are poorly visible due to crushing artifacts. (b) Anti-TTF1 immunostaining, x100 magnification: tumor cells are TTF1+. (c) HES staining, 100x magnification: invasionof the breast parenchyma by a tumor with large, atypical cells. (a) Anti-TTF1 immunostaining, x100 magnification: tumor cells invading the breast are also TTF1+. HES, hematein, eosin, saffron; TTF1, thyroid transcriptase factor 1.

Histology and immunohistochemistry confirmed breast metastasis from primary lung carcinoma, with immunostaining positive for TTF1 (Thyroid Transcriptase Factor 1) and negative for Estrogen Receptor, Progesterone Receptor and Human Epidermal Growth Factor Receptor-2 (Figure 3b and d). Additional GATA 3 immunostaining was negative.

## Differential diagnosis

A solid breast nodule in a young female patient with no medical history has a broad range of differential diagnoses: fibroadenoma, phyllodes tumor, primary breast cancer, and rarely metastasis.

## Treatment

The patient was treated by Crizotinib (Tyrosine kinase inhibitor) and responded very well within 2 months. The breast and lung lesions disappeared completely. The small cerebral lesions decreased in size.

## Outcome and follow-up

The patient is still under active follow-up. After 9 months of treatment, a centimetric pulmonary lesion reappeared in the left upper lobe which is why a second line of treatment was prescribed (Repotrectinib: another tyrosine kinase inhibitor).

## Discussion

Primary breast cancer is the most common malignancy in adult females. However, metastatic involvement of the breast is a rare phenomenon, with a frequency of approximately 0.2–1.3%.^1^ Several neoplasms have been reported to metastasize to the breast such as melanoma, lymphoma, lung cancer and ovarian cancer. There are few reported cases of breast metastasis from primary lung carcinoma in the literature. Breast metastases usually have no typical features and can be easily mistaken for benign nodules although they can present as firm masses with irregular borders.^2^

The most common sites of lung cancer metastasis are the brain, the bones, and the adrenal glands, with the potential to metastasize to nearly any organ.

The main differential diagnosis was synchronous primary tumors of the lung and breast *vs* breast metastasis or a benign breast nodule posing a diagnostic dilemma. The common ultrasound features of primary breast cancer are indistinct, irregular, not well-circumscribed, hypoechoic and posterior acoustic shadowing associated with microcalcifications. The common ultrasound features of breast metastasis from lung cancer are irregular, hypoechoic and parallel masses without posterior enhancement.^3^ The atypical feature for a benign lesion such as fibroadenoma was the numerous microlobulations.^4^

The contribution of immunohistochemistry is helpful, and one reliable immunostain is the TTF1 (thyroid transcriptase factor 1) positive in 73 to 88% of cases of lung cancer.^5^ Nevertheless, a study done by Judith Robens et al. Reported a 2,4% TTF1 positivity in primary breast cancer^6^ which is why a positive TTF1 alone cannot exclude the possibility of a breast origin. An additional immunostain GATA 3 was done and turned out negative. This immunostain is sensitive for breast and urothelial carcinoma and triple negative breast cancer. Furthermore, a positive GATA 3 staining is seen in less than 10% of lung adenocarcinoma.^7,8^ The context and the negativity of estrogen, progesterone, Her-2 receptor and GATA 3 staining led us to the diagnosis of breast metastasis from lung cancer.

Similar to almost 80% of the cases reported since 2000, the breast lesion was homolateral to the lesion of the lung.^9^ Huang et al made the hypothesis of direct invasion of the parietal pleura and the chest wall lymphatic vessels by lung cancer, followed by an invasion to ipsilateral axillary lymph nodes and retrograde lymphatic spread to the breast.

## Conclusions

Lung cancer can metastasize almost everywhere in the body. Breast metastasis of lung cancer are rare, and the differential diagnosis can sometimes be challenging with benign breast nodules being more frequently encountered in the young female population. In a context of cancer, it is important to assess the benign or malignant nature of a breast nodule since it impacts the staging and the treatment options even if the ultrasound features may seem benign. For this reason, biopsy of breast nodules when encountered should be advised as part of the metastatic workup for lung cancer.

## Learning points

- Physicians should be aware of the existence of breast metastasis.
- Breast metastases have no typical features and can present as irregular circumscribed microlobulated and hypoechoic nodules.
- Biopsy of breast lesions is advised in a context of lung cancer.
- TTF1 immunostaining is highly specific and additionnal immunostains such as GATA 3 can be used to confirm the origin of the lesion.
